# Surgical removal of calcified amorphous tumor localized to mitral valve leaflet without mitral annular calcification

**DOI:** 10.1186/s40792-015-0040-6

**Published:** 2015-05-01

**Authors:** Shinya Masuda, Naotaka Motoyoshi, Koki Ito, Yukihiro Hayatsu, Masatoshi Akiyama, Shunsuke Kawamoto, Yoshikatsu Saiki

**Affiliations:** Division of Cardiovascular Surgery, Tohoku University Graduate School of Medicine, 1-1 Seiryocho, Aoba-ku, Sendai, 980-8574 Japan

**Keywords:** Cardiac calcified amorphous tumor, Mitral valve leaflet, Glutaraldehyde-treated autologous pericardium

## Abstract

A cardiac calcified amorphous tumor (CAT) localized to the mitral valve leaflet without mitral annular calcification (MAC) is a rare entity. We report a case of a 69-year-old woman with such a condition, who underwent successful excision of the tumor and mitral valvuloplasty using a glutaraldehyde-treated autologous pericardium. During 38 months of follow-up, no recurrence of a cardiac mass has been recognized. This report addresses questions on the surgical indication for CAT, particularly in cases without MAC, and reviews CATs of the mitral valve.

## Background

A cardiac calcified amorphous tumor (CAT) is a non-neoplastic cardiac mass that includes nodular calcium deposits, chronic inflammatory cells, hyalinization, and degenerating blood elements [1]. Most cardiac CATs originating from the mitral valve are related to extensive mitral annular calcification (MAC) [2-12]. Some CATs have demonstrated a diffuse calcification of the left ventricular myocardium and occasionally involved the mitral annulus, mitral chordal apparatus, and papillary muscle [11,12]. CAT localized to the mitral valve leaflet without MAC is a rare condition.

We discuss a surgical indication for CAT on the mitral valve with specific consideration for the presence and absence of MAC after reviewing the 18 previously reported cases.

## Case presentation

The patient was a 69-year-old woman whose abnormality was initially identified by chest radiography at a local hospital in 2008. At that time, transthoracic echocardiography revealed a calcified and immobile mass on the posterior mitral valve leaflet. She was referred to our hospital for evaluation of the mass. Her past history was significant with pulmonary tuberculosis at the age of 6 years and systemic hypertension. However, there was no history of infectious endocarditis or chest trauma. Any thrombus formation due to a hypercoagulable state was not recognized. Differential diagnoses included calcified cardiac fibroelastoma and myxoma. We offered her a surgical treatment option because of the potential risk of a thromboembolic event. However, she refused the surgical intervention. She was managed on an outpatient basis and periodically underwent transthoracic echocardiographic evaluations every 6 months.

**Figure 1:**
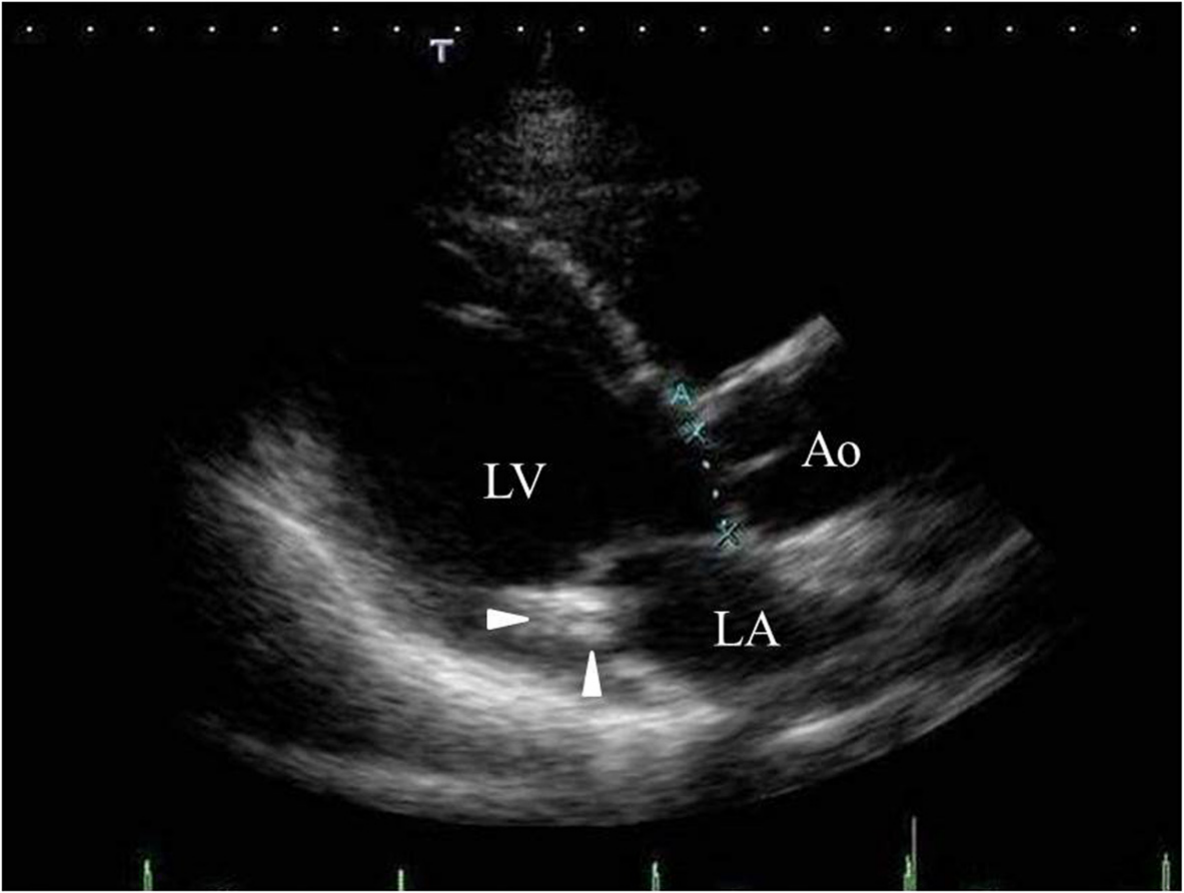
Two-dimensional transthoracic echocardiogram. Parasternal long-axis view demonstrating the high echoic mass (arrow heads) attached to the posterior mitral valve leaflet. LV, left ventricle; LA, left atrium; Ao, aorta.

**Figure 2:**
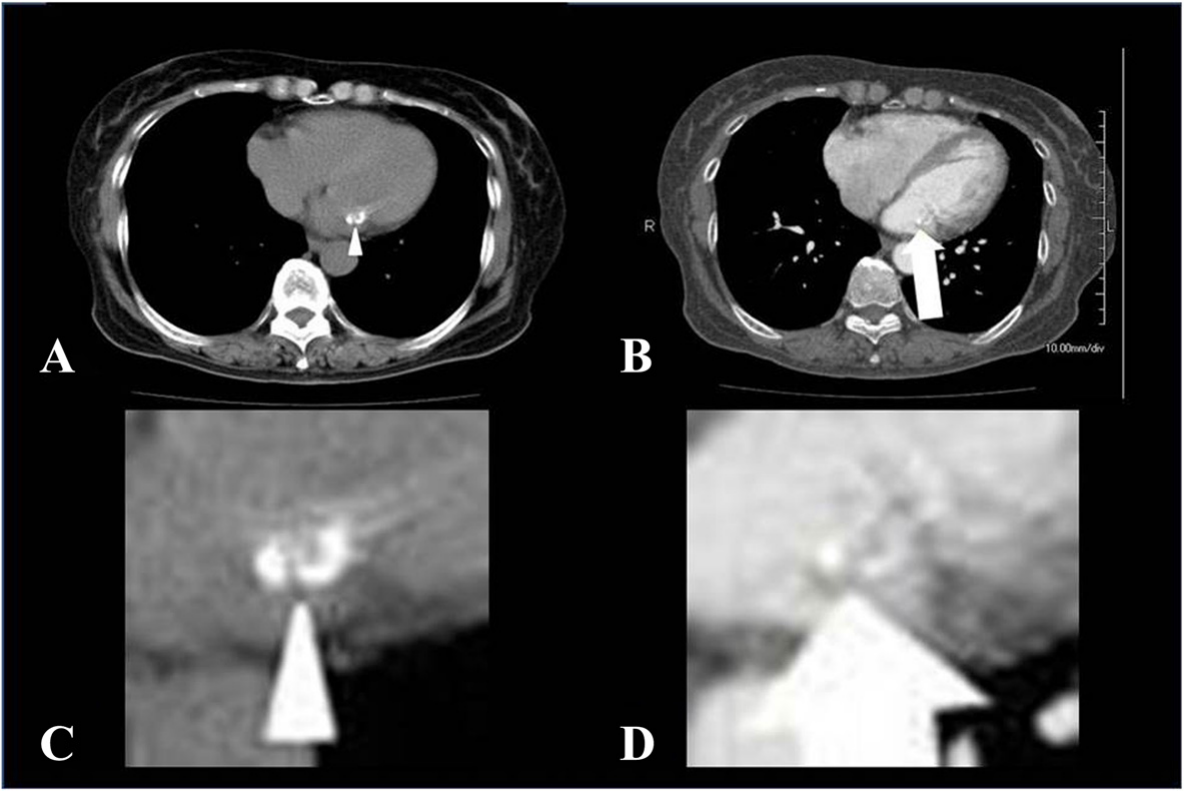
Computed tomograms (CT) of the heart. CT of the heart without contrast medium revealed a calcified mass with a mixture of high and low density area along the posterior mitral leaflet (arrow head) **(A)**, and another computed CT with contrast medium demonstrated a hypodensity central part of the mass (arrow) **(B)**. The zoomed CT of the heart without contrast medium revealed a calcified mass with a mixture of high- and low-density area along the posterior mitral leaflet (arrow head) **(C)**, and another zoomed CT with contrast medium demonstrated a hypodensity central part of the mass (arrow) **(D)**.

The patient claimed to have chest discomfort in March 2011 and was admitted to our hospital in October 2011. A transthoracic echocardiogram showed that the calcified mass measuring 19 × 8 mm was located between the P2 and P3 areas of the posterior mitral valve leaflet. There was no mitral regurgitation, and her ejection fraction was 67%. There were no findings suggestive of infectious endocarditis (Figure 1). On cardiac catheterization, the coronary arteries were intact without an obvious thrombo-occlusive lesion. Computed tomograms (CT) confirmed the severe calcified mass on the posterior mitral valve leaflet (Figure 2). Laboratory examinations were unremarkable.

Because there was potential risk of thromboembolism, surgical intervention was indicated for the calcified mass or tumor on the mitral valve. Definitive diagnosis was also expected after surgical excision of the mass.

The patient underwent surgical removal of the mass with the aid of cardiopulmonary bypass. The mitral valve was inspected through a right-sided left atrial incision. A round mass was originating from the P2 area and measured 8 mm in diameter. The calcified process was localized to the posterior leaflet itself. The entire mass was excised and submitted for histopathological examination. The defect on the posterior leaflet of the mitral valve was patched with a glutaraldehyde-treated autologous pericardium.

No regurgitation was detected after the procedure. Weaning from the cardiopulmonary bypass was uneventful.

**Figure 3:**
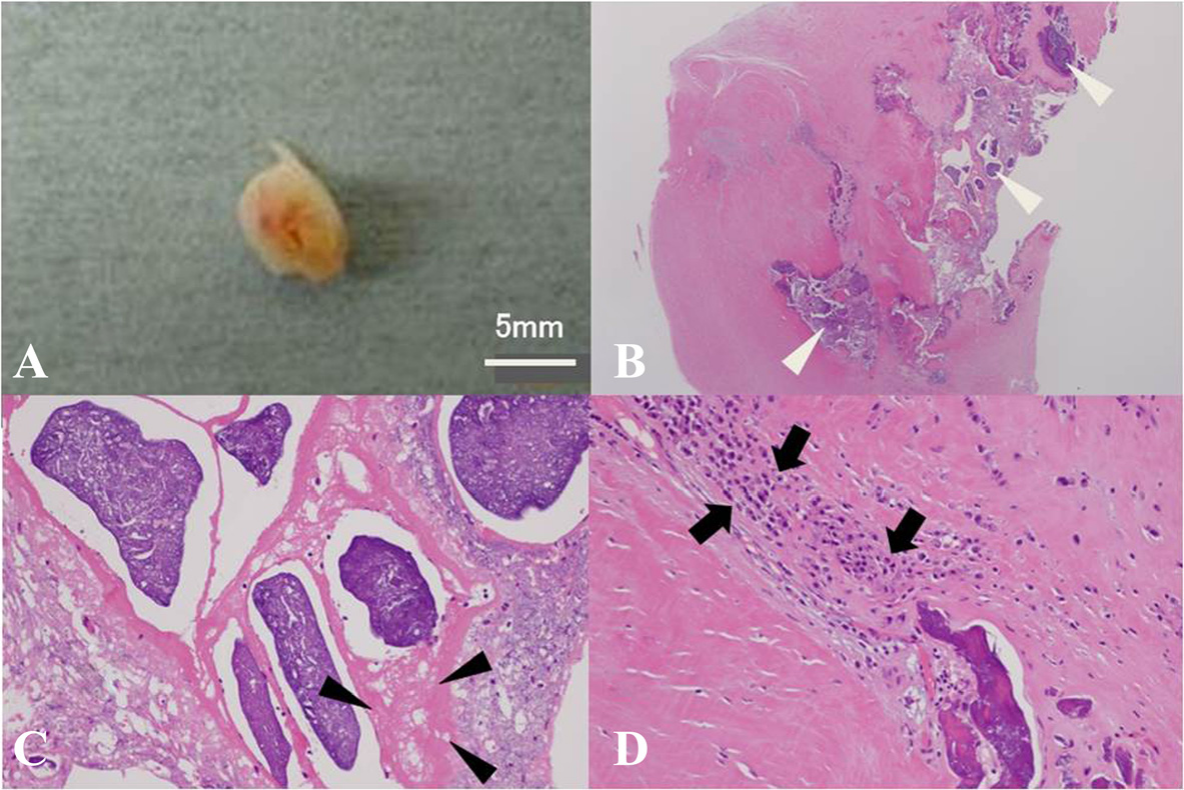
Macroscopic appearance and histological examination of the calcified mass excised from the posterior mitral valve leaflet. Macroscopic appearance of the calcified mass excised from the posterior mitral valve leaflet **(A)**. Histological examination revealed multiple nodular calcifications (white arrow heads) surrounded by fibrous connective tissue (hematoxylin-eosin stain, original magnification × 2) **(B)**, dense fibrin (black arrow heads; hematoxylin-eosin stain, original magnification × 20) **(C)**, and focal infiltration of inflammatory cells, predominantly plasma cells and lymphocytes, adjacent to the calcification (black arrows; hematoxylin-eosin stain, original magnification × 20) **(D)**.

Grossly, the mass was white and measured 9 × 5 × 2 mm. Histological examination revealed nodular calcification in the fibrous connective tissue with dense fibrin and mixed inflammatory infiltration, predominantly with plasma cells and lymphocytes. There were no identifiable myxoma cells or malignant cells. Consequently, a definitive diagnosis of cardiac CAT was made (Figure 3).

The patient recovered uneventfully and was discharged without any adverse events. No recurrent cardiac mass has been detected at 38 months after the surgery.

## Discussion

Forty-four cases of cardiac CAT have been reported previously. As a significant feature, CATs were originating from the mitral valve in 18 of these cases (Table 1) [1-15]. Fourteen were related to extensive MAC [2-12], two of which demonstrated diffuse calcification [11,12]. CATs localized to the mitral valve leaflet, without MAC, are rare [1,13-15]. The present case appears to be the fifth case of that kind.

**Table 1:** **Review of CAT on MV**

**Case number**	**Authors (year)**	**Age (year)/gender**	**Manifestation**	**Location of tumor**	**MAC**	**CRF**	**HD**	**Treatment**	**Comment**
1	Kawata (2013) [2]	59/M	None	MA	+	+	+	Resection	MAC-related CAT
2	Fujiwara (2012) [3]	58/M	None	MA	+	+	+	Resection + MVP	MAC-related CAT
3	Fujiwara (2012) [3]	65/M	None	MA	+	+	+	Resection	MAC-related CAT
4	Nishigawa (2012) [4]	78/F	None	MA	+	-	-	Resection	MAC-related CAT
5	Kubota (2010) [5]	64/F	None	MA	+	+	+	Resection + MVR	MAC-related CAT
6	Kubota (2010) [5]	44/M	None	MA, LV, LA, papillary muscle	+	+	+	Resection	MAC-related CAT
7	Mohamedali (2014) [6]	69/F	SOD	MA, LV outflow tract	+	+	-	Resection	
8	Yamamoto (2014) [7]	82/F	Heart failure	MA, LV outflow tract	+	-	-	Resection + AVR	
9	Inamdar (2008) [8]	85/F	Fatigue	MA	+	+	-	Resection	
10	Poh (2007) [9]	85/F	CVA	MA	+	-	-	Resection	
11	Morishima (2006) [10]	68/M	Dyspnea	MA	+	+	+	Resection	
12	Morishima (2006) [10]	65/M	Dyspnea	MA	+	+	+	Resection + MVR	
13	Vlasseros (2011) [11]	65/F	Visual disturbance	MA, chordae tendinae	+	-	-	Resection + MVR	Retinal arterial emboli
14	Habib (2010) [12]	58/F	Recurrent VTs	MA, papillary muscle	+	-	-	Antiarrhythmic therapy	Placement of an ICD
15	Reynolds (1997) [1]	48/F	CVA	MV	-	-	-	Resection	
16	Hussain (2014) [13]	69/F	Palpitation	AML	-	-	-	Resection + MVP	
17	Nishimori (2014) [14]	68/M	Leg pain	PML	-	+	+	Resection	PD
18	Greaney (2011) [15]	69/F	Heart failure, stroke	LV, base of MV	-	-	-	Resection	
19	Present case (2015)	69/F	Chest discomfort	PML	-	-	-	Resection + MVP	

CAT, calcified amorphous tumor; MV, mitral valve; MAC, mitral annular calcification; CRF, chronic renal failure; HD, hemodialysis; MA, mitral annulus; MVP, mitral valve plasty; MVR, mitral valve replacement; LV, left ventricle; LA, left atrium; SOD, shortness of breath; AVR, aortic valve replacement; CVA, cerebrovascular accident; VT, ventricular tachycardia; ICD, implantable cardioverter defibrillator; AML, anterior mitral valve leaflet; PML, posterior mitral valve leaflet; PD, peritoneal dialysis.

Our literature review revealed that most patients with MAC-related CATs are on hemodialysis. It is therefore possible to preoperatively determine whether a calcified intracardiac mass of the mitral valve is CAT by the presence of extensive MAC in end-stage renal disease patients. Our patient did not have end-stage renal disease, and there was no history of endocarditis or thrombus formation. It was impossible to distinguish a calcified intracardiac mass of the mitral valve from calcified cardiac myxoma, fibroma, fibroelastoma, and caseous MAC [16,17] by diagnostic imaging. The caseous MAC is a calcified mass along the mitral leaflet. Its diagnostic image is very similar to that of CAT. It has been clearly characterized using CT in recent years. Non-contrast CT typically shows a large calcified mass in the region of the posterior mitral annulus extending to the adjacent mitral valve. On contrast medium-enhanced CT, the central part appears less hypodensed due to the caseous toothpaste-like material. In our case, CT of the heart without contrast medium revealed a calcified mass with mixture of high and low density along the posterior mitral leaflet, and enhanced CT showed the hypodensed central part. Intraoperative and histological findings were negative for a caseous toothpaste-like necrosis. The surface of the tumor was unexpectedly smooth in contrast to the preoperative CT findings. The calcified lesions were well capsulized.

Operative and histological findings of the small portion of the posterior leaflet was quite normal and negative for any sign of previous injury, formation of thrombus, presence of fungus body, and caseous necrosis. The mechanism which the CAT without MAC arose was not defined. Since the lesion was not diffuse but rather discrete, we may speculate that its etiology is not inherent. Based on these preoperative diagnostic findings, we could not exclude a risk of thrombus formation and caseous necrosis occurring during the course of this pathology. Hence, certain level of risk of thromboembolism was not neglected prior to surgical intervention. Regardless of the benign or malignant nature of a cardiac mass, an excision of the lesion is important to avoid the potential risk of embolization [3,5,8-10,18] and to determine the accurate diagnosis and therapy [18,19].

In terms of the size, we are unable to define the size criteria toward surgical intervention for calcified tumor on the mitral valve. Although it has been known that a risk of thromboembolism increases when the size of vegetation exceeds 10 mm, this size criteria should apply to the case with vegetation associated with infective endocarditis. We presume that it is not easy to decide surgical indication for CAT by the size of the mass. We need to accumulate more number of cases with cardiac mass of suspected CAT.

CAT localized to the mitral valve leaflet without MAC in our case was unique, and its etiology has not been determined. In general, CAT is pathologically diagnosed after surgical excision. The prognosis of a patient with a cardiac mass is unknown unless surgical excision is performed. This patient had chest discomfort. However, she had no signs of heart failure or arrhythmia. We offered her a surgical treatment option on an occasion of her first visit considering certain risk of thromboembolism and for the purpose of diagnosis. However, she declined a surgical intervention. She was managed on an outpatient basis. She started to worry about her future health condition especially after the Great East Japan Earthquake. Although the reason for her chest discomfort was not clear, it certainly enhanced her treatment which might help an avoidance of stoke or other thromboembolic event.

When a calcified tumor localized to the mitral valve leaflet with MAC is immobile, it can be conservatively observed as CAT. In contrast, as observed in our case, if a calcified mitral tumor without MAC cannot be distinguished from a calcified cardiac myxoma, fibroma, fibroelastoma, and caseous MAC by conventional diagnostic imaging, we should consider surgical intervention. Before deciding a surgical indication for the calcified tumors localized to the mitral valve leaflet, the specific determination of the presence or absence of MAC help to define the surgical indication.

## Conclusions

We reported a rare case of CAT localized to the mitral valve leaflet without MAC. A literature review revealed that the presence of MAC is suggestive of CAT on the mitral valve. However, even with the absence of MAC, CAT remains a potential diagnosis, and surgical intervention is still considered.

## Consent

Written informed consent was obtained from the patient for publication of this case report and any accompanying images. A copy of the written consent is available for review by the Editor-in-Chief of this journal.
